# Supplementary material for: Patterns of Intron Gain and Loss in Fungi
Source: PLoS Biol. 2004 Nov 30;2(12):e422. doi: 10.1371/journal.pbio.0020422 (PMC532390; doi:10.1371/journal.pbio.0020422)
Supplement: Table S1 — Also available at http://genes.mit.edu/NielsenEtAl/. (4.3 MB ZIP). [file pbio.0020422.st001.zip › NielsenEtAl/html/639.html]

AN2733.1.NCU02771.1.MG01622.1.FG01361.1


```
 CLUSTAL W (1.82) Multiple Sequence Alignments - Introns Inserted


Sequence 1: AN2733.1	378 aa
Sequence 2: FG01361.1	366 aa
Sequence 3: MG01622.1	367 aa
Sequence 4: NCU02771.1	390 aa
Alignment Length: 413 aa
Number Identitical Residues: 220 aa
Alignment Score (without introns) 10696


MG01622.1 	-----------------------------MGHTFEPLKNDLVIRTAWG1QKVER~PPIWI
NCU02771.1	MAAASLCLLSLSTALVAWYLHNKSTNRHKMASQFPPLKNDLLLRAAR-~----A1P---L
FG01361.1 	-----------------------------MTQDFAPLKNDLLLRTAWG1QTVER~PPMWV
AN2733.1  	-----------------------------MTQ-FAPLKNDLLLRAAWG1DKVHR~PPIWV
          	                             *   * ******::*:* . ..    *.  :

MG01622.1 	MRQA1GRYLPEYHEAKGNRDFFECCRDPEVASTLTLQPIDRFDGLLDASIIFSDILVIPQ
NCU02771.1	ILTA~GRYLPEYHEAKGGRDFFEVCRNPEIASTLTLQPIERYAGLIDAAIIFSDILVIPQ
FG01361.1 	MRQA1GRYLPEYHEAKGNRDFFECCRDPEVATEITLQPVRRFAGLIDAAIIFSDILVIPQ
AN2733.1  	MRQA1GRYLPEYHEAKGNRDFFECCRDPEIASTITIQPVDRYEGLIDAAIIFSDILVIPQ
          	:  * ************.***** **:**:*: :*:**: *: **:**:***********

MG01622.1 	AMGMVVEMVDKKGPHFPNPLQSPDDGQYAEVLARDVDVSKELDYVYKAITLTRKKLQGRV
NCU02771.1	AMGMTVEMVDKKGPHFPNPLRSPSDGQYAELMKRDVDVAKELDYVYKAITLTRKKLDGRV
FG01361.1 	AMGMTVEMVDKKGPHFPEPLKSPDDGQYGQVLAKDVDVAVELGYVYDAITLTRQKLDGQV
AN2733.1  	AMGMTVEMVDKKGPHFPEPLRSPTDGQYEKVMARDVDVKAELDYVYKAITLTRTKLNGRV
          	****.************:**:** **** ::: :****  **.***.****** **:*:*

MG01622.1 	PLIGFCGAPWTLFCYMVEGGGTKLFIQSKKWIFRHPEESKKMLQKIAELCVEYLALQVQA
NCU02771.1	PLFGFTGAPWTLLCYMVEGGGTKLFKEVKTWIYKYPEETKALLQKISELCVEYLALQVKA
FG01361.1 	PLIGFCGAPWTLFCYMVEGGGTKLFAQVKTWIYKYPEEAKKLLSKIADICVEHLALQVKA
AN2733.1  	PLIGFCGAPWTLLCYMVEGGGSKMFIQTKTWIYKYPKESQALLQKIAEICVEYLALQVAA
          	**:** ******:********:*:* : *.**:::*:*:: :*.**:::***:***** *

MG01622.1 	GAQ0LVQVFDSWAGELSPASFEQFSAPYLRYISENLPKRLKELSLDPVPMIVFPKGAWFA
NCU02771.1	GAQ0IVQVFDSWAGELSPSSFKTFSQPYLTYIAKHLPLRLKEMGLEPVPMVVFPKGAWYA
FG01361.1 	GAQ0LVMVFDSWAGELGPASYRQFSEPYLKQIAEKLPAKIQSLGLEKVPMTVFPKGAWYA
AN2733.1  	GAQ~LVQVFDSWAGELSPASFQDFSLPYLRHISANLPRRLKEMGLEPVPMTVFAKGAWYA
          	*** :* *********.*:*:. ** ***  *: :** :::.:.*: *** **.****:*

MG01622.1 	LEDCCAMGYNVVGLDWLYSPAKANQVRGDREIVLQGNADPGVLYGTKDAITAAVKDMVEG
NCU02771.1	LDAACDMGYNVVGLDWLHDPAEAVKVVGDRPMVLQGNADPGVLYGSHEAITKVVEEMVQG
FG01361.1 	LDSACNLGYNVVGMDWLHDPKEAVKIRGDRNIVFQGNADPGVLYGTKEAITKAVEEMVEG
AN2733.1  	LEDLCESGYNVVGLDWLHDPAEAFKIANGR-VTIQGNADPGVLYGGREAIKQAVETMVAG
          	*:  *  ******:***:.* :* :: ..* :.:*********** ::**. .*: ** *

MG01622.1 	FDWKEKKKGWIVNLGH---------------G1ITPLVNPDDLKFYLQEIHRQTKD--
NCU02771.1	FDWHGRHKGWIVNLGH---------------G~ITPFVNPDDLKFFFEEIHRLTKTKA
FG01361.1 	F--WVGDKGWIANLGH---------------G1ITPGVNPDHLKHYFEEIHRLTKKN-
AN2733.1  	F--KKGKQGWIANLGHGMLFIRLYHADANFLG~VTPFVNPDDLKFYFEEIHRLTAD--
          	*     .:***.****.        :.:.  * :** ****.**.:::**** *
```
